# Cognitive behavioural therapy for older adults with depressive symptoms living in residential aged care: A pilot study of a systemic treatment model

**DOI:** 10.1111/papt.70075

**Published:** 2026-04-30

**Authors:** Joanna M. Waloszek, Sofie Dunkerley, Sunil Bhar, Deborah Koder, Tanya E. Davison, Penelope Schofield, Steven Quinn, Julie Ratcliffe, Mark Silver, Jennifer Linossier, Rebecca Collins, Rachel Milte

**Affiliations:** ^1^ Department of Psychological Sciences Swinburne University of Technology Hawthorn Victoria Australia; ^2^ Silverchain Melbourne Victoria Australia; ^3^ Iverson Health Innovation Research Institute Swinburne University Melbourne Victoria Australia; ^4^ School of Computing, Engineering and Mathematical Sciences La Trobe University Bundoora Victoria Australia; ^5^ Sir Peter MacCallum Department of Oncology The University of Melbourne Parkville Victoria Australia; ^6^ Caring Futures Institute Flinders University Adelaide South Australia Australia

**Keywords:** ageing, anxiety, depression, residential aged care, treatment

## Abstract

**Objectives:**

Older adults in residential aged care have disproportionately high rates of depression compared to community settings, but access to mental health services is poor. The study aimed to evaluate the feasibility and acceptability of a novel outreach student‐delivered systemic therapy program (ELders AT Ease [ELATE]) for addressing depressive symptoms in residential aged care.

**Design:**

Single‐arm pre‐ and post‐intervention design.

**Methods:**

The program integrates evidence‐based cognitive, behavioural and reminiscence techniques. Sixteen individual face‐to‐face therapy sessions with residents over 5 months were delivered by supervised postgraduate psychology students on placement. Family and staff were invited to join resident therapy sessions and monthly psychoeducational group sessions. Aged care residents with depressive symptoms and no or mild cognitive impairment were recruited. Participants completed measures of depression, anxiety and quality of life at four timepoints. Program satisfaction and treatment integrity were assessed post‐treatment. Study uptake, treatment uptake and retention rates were calculated.

**Results:**

Fifty‐four residents were referred; 16 were eligible, and 15 participated (12 female, 87.3 ± 6.3 years). Overall, 14 participants completed at least 12 of 16 treatment sessions. Treatment satisfaction was high, with 90.9% of residents rating the treatment as ‘good’ or ‘excellent’. Preliminary clinical outcomes suggested an average decrease post‐treatment in depression and anxiety, with small‐to‐medium effect sizes. Staff and family session involvement was lower than expected.

**Conclusions:**

The pilot suggested that recruitment, screening and 5‐month treatment procedures were feasible and acceptable. The results warrant a larger study to examine the effectiveness of the student‐delivered outreach approach in residential aged care.

## INTRODUCTION

Depression in older adults is a common and significant problem, with global prevalence rates between 28.4% and 35.1% (Cai et al., 2023; Hu et al., 2022). Adults living in residential aged care (RAC) facilities (also known as residential aged care homes, nursing homes or long‐term care homes) have disproportionately high rates of depression compared to community settings, in Australia as well as internationally (Jongenelis et al., 2004; Tang et al., 2021, 2022; Zenebe et al., 2021). Over 50% of people who live permanently in RAC in Australia have clinically significant symptoms of depression compared with 10% of older adults in the general population (Australian Institute of Health Welfare, 2013). Depressive symptoms can be misunderstood as a normal part of ageing in such settings, and residential care staff often lack skills to identify and refer residents to treatment services (McCabe et al., 2008). Adding to the complexity, access to mental health services in RAC is poor, mental health clinicians are largely absent and there is an over‐reliance on psychotropic medication as the primary or sole treatment strategy (Cations et al., 2022; Stargatt et al., 2017; Westbury et al., 2019).

Psychological treatments for depression such as cognitive behaviour therapy (CBT), behavioural therapy and reminiscence therapy have been found to be effective for older adults (Ji et al., 2023). However, few high‐quality studies have been conducted in RAC. A recent Cochrane review of randomised controlled trials (RCTs) found that in the last 50 years, only 19 studies had been conducted in RAC, and the quality of research was poor (Davison et al., 2024). Aged care residents are more likely to be frail and experience higher levels of cognitive impairment and chronic ill health than community‐dwelling adults. Aged care residents are also more likely to be functionally impaired and to be dependent on others for daily assistance and care, and have difficulty travelling to outpatient mental health services. Psychological treatments may need to be adapted to suit the profile of RAC residents (Chan et al., 2021).

The application of treatments that were originally designed for outpatient community‐dwelling patients to RAC populations is believed to require adaptation to include a more systemic approach (Bhar et al., 2022). Systemic approaches are those that include the patients' families and staff carers in treatment. Such approaches are typically used for individuals who are highly dependent on others for day‐to‐day care. An example of such an approach is that used by the Peaceful Mind program where families were considered instrumental co‐therapists in a program for reducing anxiety in individuals with dementia (Paukert et al., 2013). In RAC, several treatment programs have included staff as such treatment collaterals, a feature which increased their effectiveness (Cody & Drysdale, 2013). Given that nearly 50% of individuals living in such settings also have significant levels of cognitive impairment (Australian Institute of Health Welfare, 2013), the involvement of staff and family is expected to help residents with memory and attentional difficulties more successfully implement and practice therapeutic strategies.

Further, to address the shortage of mental health clinicians in RAC, a student‐delivered approach may be a viable alternative (Grealish et al., 2013). Evidence suggests that creating quality placements for clinical psychology students in RAC not only has benefits for both residents and students but also addresses the need for more mental health clinicians with clinical geropsychology competencies (Koder & Helmes, 2008).

The ELders AT Ease (ELATE) psychological intervention model was developed for the treatment of late‐life depression in RAC to address the need for a systematic approach that is potentially feasible and scalable given shortages in the mental health workforce. The program is manualised and utilises the clinical psychology student workforce to deliver the program face‐to‐face to residents. The treatment integrates evidence‐based cognitive, behavioural and reminiscence techniques in individual therapy sessions with residents over 5 months. The treatment model is systemic in nature by collaborating with, supporting, and training family carers and RAC staff in managing depressive symptoms.

This current study evaluated the feasibility and acceptability of the ELATE treatment program for older adults with depressive symptoms who were living in RAC. The study focused on examining retention and treatment completion rates, the extent to which protocols were acceptable to participants and staff, and whether treatment was delivered as intended.

## MATERIALS AND METHODS

### Setting

204 RAC facilities within a 15 km radius of Hawthorn, Victoria, were invited to the study by mail. The first five facilities that responded with interest and signed a research agreement were included.

### Design

This study used a single‐arm pre‐ and post‐intervention design. Treatment was delivered face‐to‐face in the RAC facility by trained postgraduate psychology students on placement. Clinical outcomes were assessed at baseline, mid‐treatment (2.5 months post‐baseline), post‐treatment (5 months post‐baseline) and at 3‐month follow‐up (8 months post‐baseline). Assessments were conducted as semi‐structured interviews with residents by trained research assistants. The project was approved by the Swinburne University Human Research Ethics Committee (Ethics ID: 1170).

### Participants and recruitment procedure

**FIGURE 1:**
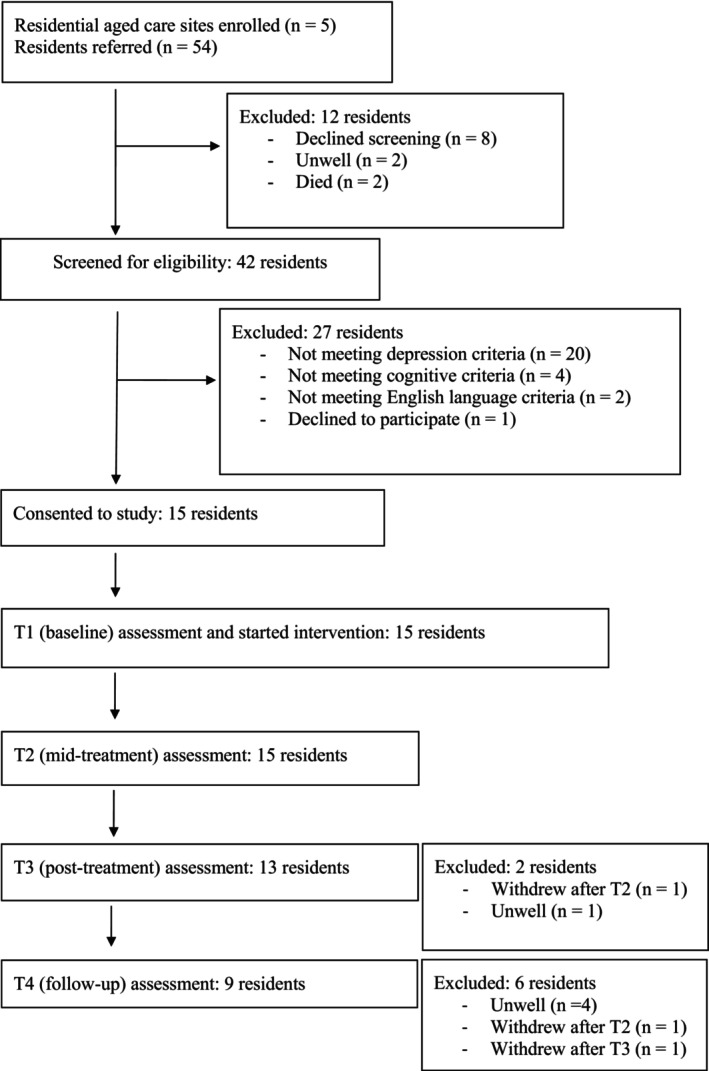
Recruitment summary.

Recruitment occurred between August and November 2019. A flow diagram of participant recruitment is presented in Figure 1.

The RAC facility manager (or their designee) at each facility was invited to refer residents they considered were likely to be depressed and cognitively intact. The manager was permitted to use various methods to select potentially suitable residents. First, the manager could select residents who in their subjective opinion were depressed and had no more than mild cognitive impairment. Second, managers could select residents who scored ≥9 on the Cornell Scale for Depression in Dementia (CSDD) (Alexopoulos et al., 1988) and ≤9 on the Psychogeriatric Assessment Scale – Cognitive Impairment Scale (PAS‐CI) (Jorm et al., 1995), as recorded in their case files over the last 6 months. Third, they could survey their pool of residents using the Patient Health Questionnaire‐2 (PHQ‐2) (Kroenke et al., 2003) to establish current levels of depressive symptoms. With the residents' verbal consent, the managers provided the residents' names to researchers. The main staff contact person or ‘key staff member’ at each facility who assisted the researchers was invited to give feedback after the program.

Research assistants screened referred residents. Screening was conducted face‐to‐face, on site at the RAC facility with the resident alone.

Inclusion criteria: Residents were eligible if they were aged 65 years or older and had significant depressive symptoms (scored at least 6 on the 15‐item version of the Geriatric Depression Scale, GDS‐15 or at least 3 on the 2‐item version of the Patient Health Questionnaire, PHQ‐2). Exclusion criteria: Residents were ineligible if they (1) were too unwell to participate in study tasks; (2) were unable to communicate in English or (3) had moderate or severe cognitive impairment (scored below 15 on the Montreal Cognitive Assessment, MOCA, (Nasreddine et al., 2005) or below 21 on the Standardised Mini Mental State Exam, SMMSE (Molloy & Standish, 1997)).

Eligible residents were invited to provide written informed consent. They were also invited to nominate a family member and staff member to assist them in treatment or to defer to the facility manager's nomination of such supports.

### Therapists

The intervention was delivered by four postgraduate students on clinical placement while completing their masters or doctorate studies in clinical psychology. These students, referred to as mental health trainees (MHTs), were selected if they (a) had some experience delivering one‐to‐one counselling to clients and (b) were positively evaluated as reflective and empathic by a placement interview panel of three clinicians. Students were not required to have clinical experience in aged care or with older adults.

Before treating residents, MHTs completed 2 weeks of purpose‐developed online training in mental health and aged care and attended a full day training session with senior clinical psychologists. The online training comprises self‐guided readings and videos, as well as learning activities. The training session included discussions about common clinical presentations in RAC and expected professional conduct (e.g., confidentiality, risk management). The session also included an overview of the research protocol and intervention manual, as well as training on delivering prescribed intervention strategies. MHTs received fortnightly individual and group supervision. Clinical supervisors were senior clinicians experienced in aged care and mental health care who supported MHTs by fostering reflection, teaching clinical skills and providing feedback on clinical approaches.

### Intervention

The intervention, called ELders AT Ease (ELATE) involved three components. The first component comprised 16 weekly face‐to‐face individual sessions between the MHT and residents over 22 weeks (allowing for missed weeks due to ill health or other commitments). Initial sessions (1–4) represented the engagement phase, where the focus was on the development of rapport with the resident. The intermediate sessions (5–14) represented the treatment phase, where MHT and client discussed the presenting issues, background of the problem and the impact of the problem on the client's daily functioning. The final phase (15–16) involved a review and consolidation of strategies for extending and/or maintaining treatment gains. The treatment protocol was manualised, but MHTs were permitted to sequence and tailor techniques according to the cognitive and physical capacity of the resident. Each session was planned to last 30–60 min, depending on the needs and attention span of the client. Nominated family, friends and staff were encouraged to join sessions and to help the resident engage in therapeutic activities between sessions. A summary of each module is presented in Table 1. These sessions were recorded to assess treatment integrity.

**TABLE 1:** Resident treatment modules.

Module	Aim	Sessions
1. Introduction	Orient resident to therapy and establish therapy goals	1, 2
2. Reminiscence	History taking with a reminiscence approach. Address coping strategies and encourage discussion of positive aspects of life history	3, 4, 5
3. Behavioural activation	Introduce behavioural methods such as activity scheduling	6, 7, 8, 9
4. Anxiety management	Provide rationale for how anxiety develops and is maintained. Introduce relaxation training	10, 11, 12
5. Cognitive restructuring	Introduce structured cognitive therapy techniques. Identify unhelpful beliefs and apply coping self‐talk to common situations in residential aged care facilities	13, 14, 15
6. Relapse prevention	Address relapse prevention strategies in creating wellness plans	16

The second component comprised five 2‐h psychoeducation group sessions with family and friends of the residents, conducted monthly. Groups were open to all family and friends associated with residents in the facility, not just those of study participants. Groups were facilitated by the MHT and accompanied by a clinical supervisor. Sessions were manualised and designed to provide opportunities for peer support, sharing of resources and education on how to support residents. Group participants were also provided with a booklet on self‐care methods and strategies for caring for others with anxiety and depression (Beyondblue., 2009).

The third component comprised five 2‐h psychoeducation group sessions with facility staff, also conducted monthly, by an MHT and a clinical supervisor. In session 1, staff were provided training on symptoms of depression and anxiety, and information about ELATE. Sessions 2–5 focused on strategies for supporting the emotional wellbeing of residents at the facility. Groups were open to all staff at the facility. See Tables S1 and S2 for a summary of family and staff group sessions.

### Data collection

Clinical outcome questionnaires were completed as semi‐structured interviews with residents by trained research assistants on site at the RAC facility. These assessments were conducted at four timepoints: baseline (T1), mid‐treatment (2.5 months post‐baseline, T2), post‐treatment (5 months post‐baseline, T3) and at 3‐month follow‐up (8 months post‐baseline, T4). Researchers also phoned residents and key staff members at post‐treatment to gauge program satisfaction. Further, at the end of placement, MHTs self‐reported on treatment adherence and clinical supervisors determined therapist competency by rating a random 10% of recorded intervention sessions and completing a checklist.

### Measures

#### Feasibility

Feasibility was assessed by (a) study uptake and retention rates, (b) treatment uptake and retention rates and (c) treatment integrity (therapist competency and treatment adherence).

Study uptake rate was defined as the proportion of eligible residents who consented to participate in the study. We considered an uptake rate of 70% to be acceptable, following a review of treatment studies in aged care that found an average uptake rate of 72.9% Chan, Bhar (Chan et al., 2021). Study retention rate was defined as the proportion of participants who completed assessments at baseline and post‐test 5 months later (i.e., the primary endpoint).

Treatment uptake rate was defined as the number of eligible participants who attended at least 1 treatment session. Treatment uptake for family and staff was defined as the proportion of participating family and staff attending at least one psychoeducation group session and at least one joint session with the resident. Treatment retention was defined as the proportion of residents attending at least 12 of 16 sessions, a benchmark similar to those of other studies (Blandy et al., 2015; Bryant et al., 2020). Family and staff were considered to be retained in treatment if they attended at least 4 group sessions and joined at least 3 sessions with the resident.

Treatment integrity (treatment adherence and therapist competency) was measured by the Treatment Integrity Checklist (TIC) and Cognitive Therapy Rating Scale (CTRS) (Young & Beck, 1980). The TIC was a purpose‐built scale comprising 36 items that assessed MHT's adherence to the treatment manual and their competency in delivering the modules. The scale comprised 6 sections, corresponding to the 6 treatment modules. Each section comprised 4–9 items describing module activities. Adherence was measured by asking the MHT to state whether each activity in the treatment module was conducted (Yes/No). An MHT was considered adherent to a module if, according to their ratings, they completed 80% of activities for a module.

Therapist competency in delivering treatment was rated in two ways. First, to evaluate the competency of the MHT in executing treatment activities, the MHT's clinical supervisor rated the student on how well an activity was conducted using the TIC (1 = not very well to 5 = extremely well) based on their review of session audio recordings and on individual supervision sessions. Second, to evaluate the competency of the MHT in delivering treatment in line with CBT principles, two clinical supervisors independently completed the CTRS based on their reviews of 21 audio recorded sessions. The CTRS is an 11‐item rating scale that measures therapist competency in delivering CBT. A score of 40 was regarded as the threshold for demonstrating competency as per convention (Shaw et al., 1999).

#### Acceptability of study protocol

Treatment acceptability from the perspective of the resident was also assessed in two ways: the Client Satisfaction Questionnaire‐8 item version (CSQ‐8) and five open questions. The CSQ‐8 is a standardised measure which comprises 8 Likert‐scale questions. It is reported to have strong psychometric properties used to assess general satisfaction across varied health and human services (Attkisson & Zwick, 1982). The five open questions asked residents (1) whether there were any benefits to their experience (2) whether there were any downsides to their experience, (3) whether they could suggest any improvement to the program, (4) whether there were enough visits and (5) for any other feedback.

Acceptability of study protocol was assessed in two ways. Assessment acceptability was determined at the end of the T2, T3 and T4 visits. Research assistants asked residents how comfortable (1 = ‘not at all comfortable’ to 5 = ‘very comfortable’) and enjoyable (no/somewhat/yes) they found the assessment. Acceptability of the study procedures from the perspective of key staff members was obtained through a post‐program staff phone survey. Open items included the following questions (1) ‘What was the experience of the program for recipients?’, (2) ‘What was the experience like for you as study contact?’ and (3) ‘What was the general experience of having the program at the facility?’ Staff members were also asked if it was worth having the program (yes/no), whether they would recommend it to other RAC facilities (yes/no) and how satisfied they were with the program on a scale of 1 ‘not at all satisfied’ to 5 ‘extremely satisfied’.

#### Clinical outcomes

This pilot study aimed to assess feasibility and acceptability of the intervention rather than clinical effectiveness. However, clinical outcomes were assessed to provide a preliminary indication of the trajectory of symptomatic change pre‐ and post‐treatment.

Depressive symptoms were measured with the GDS‐15 (Yesavage & Sheikh, 1986) and PHQ‐9 (Kroenke et al., 2001), both of which have been validated for older adults living in RAC (Bélanger et al., 2019; Watson et al., 2009). The GDS‐15 is a 15‐item self‐report measure of depressive symptoms in older adults. Each item is scored on a binary scale (yes/no), and scores are summed to produce a total score, with a score of 6 or above usually regarded as indicative of significant depressive symptoms, with a sensitivity of 92% and a specificity of 81% for major depressive disorder (Lyness et al., 1997). The PHQ‐9 is a 9‐item measure of criteria for major depressive episode as defined by the Diagnostic and Statistical Manual, 4th edition (DSM‐IV) (Bell, 1994). Each item is scored from ‘0’ (not at all) to ‘3’ (nearly every day) (Kroenke et al., 2001). The PHQ‐9 has good psychometric properties in older adults with a cut‐off of ≥10 (Phelan et al., 2010).

Anxiety was measured with the Geriatric Anxiety Index (GAI) (Pachana et al., 2007). The GAI is a 20‐item measure of anxiety symptoms over the past 4 weeks. Internal consistency of the GAI is high: 0.91–0.93 for older adults sampled in the community and in psychogeriatric settings (Pachana et al., 2007). The GAI has high specificity (84%) and sensitivity (75%) for Generalised Anxiety Disorder (GAD).

Quality of life was measured by the Quality‐of‐Life Alzheimer's Disease Scale – Nursing Home version (QOL‐AD‐NH). The QOL‐AD‐NH is a 15‐item instrument for use with people with and without mild to moderate dementia and demonstrates satisfactory psychometric properties (Logsdon et al., 2002).

#### Data analysis

Due to the small sample size, differences between pre‐treatment (T1) and post‐treatment (T3) values were compared using effect size analyses. Hedges' g (with 90% confidence intervals) was chosen to correct for differences in group size pre‐ and post‐treatment. Where less than 20% of the items were missing on the depression, anxiety and quality‐of‐life questionnaires, item‐level data were imputed using mean substitution to calculate a total score. Effect sizes of 0.2, 0.5 and 0.8 correspond to small, moderate and large effect sizes, respectively (Cohen, 2013).

## RESULTS

### Participant characteristics

Baseline demographics of participating residents are included in Table 2. A total of 15 residents – 12 women and 3 men (aged 73–97 years) consented to the study. Most participants were born in Australia and, although two reported being bilingual, all participants answered ‘English’ to the question ‘What language do you normally speak?’

**TABLE 2:** Participant demographics.

Outcome	*N*	*M* (SD)/%
Age	15	87.3 (6.3)
Gender		
Male	3	20%
Female	12	80%
Country of birth		
Australia	10	67%
Italy	1	7%
United Kingdom	1	7%
New Zealand	1	7%
Austria	1	7%
Netherlands	1	7%
Language normally spoken		
English	15	100%
Languages spoken other than English		
German	1	7%
Dutch, German, South African, French	1	7%
Education		
Left school at ≤15 years of age	4	27%
Left school at >15 years of age	7	47%
Completed undergraduate level	3	20%
Completed postgraduate level	1	7%

Five key staff members (100% women) provided feedback about the program. Three were care managers, one was a lifestyle manager and one was a general manager. They had worked in the current facility for 1.5–24 years (mean = 10.4, *SD* = 9.9). Three staff members were aged between 25 and 54 years, one was between 55 and 64 years old and one did not report their age.

Residents were asked to nominate a staff member and family or friend to support them throughout the program. Of the 15 residents participating in the study, 11 (73%) nominated a staff member, and 12 (80%) nominated a family member or friend to support them throughout the program.

### Feasibility

Fifty‐four residents across five RAC facilities were referred to the study (Figure 1). Of these, 43 were screened and 16 (38%) were eligible. The most common reason for ineligibility was not meeting depression criteria. Recruitment at each site is presented in Table 3.

**TABLE 3:** Resident recruitment and staff/family group sessions at each Residential Aged Care Facility.

Facility	Referred	Screened	Eligible	Consenting	Eligible/screened (%)	Number of staff meetings (total attendance)	Number of family meetings (total attendance)
Site 1	6	5	2	2	40	0 (0)	0 (0)
Site 2	8	6	1	1	17	1 (2)	1 (2)
Site 3	14	9	2	2	22	1 (10)	0 (0)
Site 4	12	11	4	4	36	1 (18)	3 (26)
Site 5	14	11	7	6	64	1 (2)	1 (2)
Total	54	42	16	15	38	4 (32)	5 (30)

The study uptake rate was 93.7%. Of the 16 eligible residents screened by research assistants, 15 consented to participate. Study retention rate was 87.7%. Of the 15 participants, 13 completed both baseline (T1) and post‐program (T3) assessments.

Treatment uptake for residents was 100%. All residents attended at least 1 treatment session. Treatment retention rate for residents was 93.3%; 14 participants completed at least 12 of the 16 treatment sessions (Table 4 shows the number of treatment sessions per participant).

**TABLE 4:** Number of sessions attended by residents.

Sessions attended	Residents
16	10
14	2
13	1
12	1
8	1

In contrast, uptake by family or staff was 0%, as no family or staff joined any session with the resident and there was no site where all 5 planned group sessions were held. Table 3 shows the number of staff and family meetings in each site and the average attendance per meeting. However, missing data on attendance records prevented distinguishing between nominated staff/family of participating residents and other non‐participating staff/family at the site. Therapists reported that low attendance at joint sessions and workshops was due to residents not wishing to burden family, family being too busy to attend, COVID‐19 restrictions limiting access to facility, high staff workloads and staff/family unable to be contacted.

Of 15 residents, TIC ratings were made in relation to treatment of 14 residents who completed the treatment. Four participants were excluded from the TIC module 6 count because COVID‐19 restrictions meant the module could not be completed. Analysis of the TIC indicates that 80% of elements of the treatment manual were delivered to the residents. Summary of the TIC for the activities in each of the six modules is presented in Table 5.

**TABLE 5:** Treatment integrity checklist.

Module	Items in module	Residents (*n*)	Total items across all residents	Number of completed items	Adherence (%)	Supervisor rating
1	9	14	126	121	96	4.1
2	5	14	70	60	86	3.7
3	6	14	84	72	86	3.8
4	7	13	98	53	54	3.8
5	4	14	56	31	55	4.0
6	5	10	50	47	94	4.5

A summation of ‘Yes/No’ ratings by therapists on treatment elements showed that modules 1 and 6 had the highest adherence ratings, while modules 4 and 5 had the lowest adherence. Module 4 focused on identifying anxiety and exercises to help with anxiety. Module 5 focused on thinking and coping strategies. Both modules included items about staff/family collaboration. Two residents stated they had exercises from other sources, 4 denied experiencing anxiety and 2 did not want to involve others in therapy. In module 5, 3 participants did not identify with cognitive errors, and participants also did not wish for staff/family involvement. One activity in module 3 also asked participants to write in a diary and could not be completed by some participants due to physical difficulties with writing.

Supervisor ratings of MHT competency on the TIC were on average, 4.0 (*SD* = 0.9), suggesting that overall, MHTs executed treatment activities ‘Very Well’; however, competency ratings varied across modules and MHTs. CTRS ratings (mean = 30.6, *SD* = 6.6, range 21–44.5) suggested that competency in delivering CBT was sub‐par; of the 21 recordings reviewed, only 2 were rated as 40 or over. Most therapists scored 3 ‘satisfactory’ or above on items 3–10, which focused on patient interaction and the application of therapy. The lowest scoring items were Item 1 and Item 11 which referred to the therapist not setting an agenda or incorporating relevant homework into the cognitive therapy.

### Treatment acceptability

Eleven residents completed the CSQ‐8. The total score on the CSQ‐8 ranged from 18 to 31 (mean = 26.1, *SD* = 3.8), with an average item score of 3.2, indicating overall high satisfaction with treatment. Overall, 90.9% rated the treatment positively (‘good’ or ‘excellent’) and 81.8% would recommend the treatment to a friend.

Open‐ended reports about benefits of treatment were coded into three themes: (a) social benefits, (b) mental wellbeing and (c) cognitive benefits. With respect to social benefits, residents reported enjoying having someone to talk to, being able to talk to someone not residing or working in the facility and enjoying the regularity of the company that treatment provided. In relation to mental well‐being, residents reported that the intervention helped with relaxation and management of panic attacks, and made them feel happier, more content in their own company and more accepting of their current situation. In terms of cognitive benefits, intervention strategies were reported to help improve concentration and memory (see Table S3 for illustrative quotes).

All residents reported being either ‘comfortable’ or ‘very comfortable’ answering the assessment questions and they rated 97% of assessment visits as ‘enjoyable’ or ‘somewhat enjoyable’.

Eight of 11 residents reported no ‘downsides’ to the treatment. One resident said that it was uncomfortable to discuss difficult experiences. One resident stated that too much time was used on breathing exercises instead of conversations about life and the past. One resident explained that sometimes they would feel sad and it was hard to recall memories.

### Study acceptability

Key staff members who provided feedback about the study protocol answered ‘yes’ to the study being worthwhile at their facility and that they would recommend it. On a scale of 1 (not at all satisfied) to 5 (extremely satisfied), four staff members rated their overall satisfaction as 5 and one staff member rated it 4, with an average score of 4.8 (*SD* = 0.4).

All 5 staff members answered the open questions. They noticed that residents involved in treatment demonstrated improvement in mood. Staff noted that residents appeared to enjoy interacting and sharing life experiences with MHTs. One staff member reported developing a greater understanding of participating residents: ‘The program helped me help the resident. Instead of taking their behaviour as “that's just the person”, I could understand [the] reasons behind [it]’. Four staff said that family members were positive about the program and that it was beneficial for staff, family and residents to have someone external to the facility to talk to.

In terms of what could be improved, four staff members noted that they would have liked more residents to be involved, including those with dementia. One staff member expressed a wish to have more information about the outcomes of residents at the end of treatment. One staff member mentioned that residents were initially apprehensive because of the use of the word ‘depression’, but stated that despite this concern, the ‘outcome was great’.

### Clinical outcomes

Mean and median values of clinical outcomes are presented in Table 6. Mean depression and anxiety scores showed a decreasing trend between pre‐ and post‐treatment. The effect size was small to moderate. Average QOL‐AD‐NH remained relatively stable across the follow‐up period.

**TABLE 6:** Outcome measures.

	Baseline (T1)	Mid‐treatment (T2)	Post‐treatment (T3)	Follow‐up (T4)	Hedges' g pre–post	90% CI
GDS‐15						
Mean (SD), *n*	6.7 (3.7), 15	5.9 (2.6), 14	5.3 (3.1), 10	5.1 (2.5), 7	−0.4	(−1.1 to 0.3)
Median (Min, Max)	6 (3,14)	6 (2,9)	5 (2,10)	5 (2,9)		
PHQ‐9						
Mean (SD), *n*	11.5 (3.5), 10	6.2 (5.0), 14	8.7 (5.2), 12	6.8 (4.7), 8	−0.6	(−1.3 to 0.1)
Median (Min, Max)	11 (6,16)	8 (1,17)	11 (2,21)	9.5 (4,18)		
GAI						
Mean (SD), *n*	7.9 (5.6), 15	6.8 (6.1), 15	6.4 (5.2), 13	5.7 (7.0), 9	−0.3	(−0.9 to 0.3)
Median (Min, Max)	7 (0,18)	5 (0,19)	5.5 (0,16)	3 (0,20)		
QOL‐AD‐NH						
Mean (SD), *n*	39.3 (5.2), 15	39.1 (4.8), 15	38.2 (6.1), 13	40.1 (6.3), 9	−0.2	(−0.8 to 0.4)
Median (Min, Max)	39 (28,48)	40 (30,49)	40 (31,47)	40 (29,50)		

## DISCUSSION

This pilot provided evidence of the feasibility and acceptability of the ELATE program. The program had high uptake and retention rates for the study overall as well as for treatment. On average, therapists delivered 80% of treatment activities and demonstrated satisfactory competency in delivering treatment activities according to supervisor ratings. Residents were highly satisfied with both treatment and assessment procedures. Residents rated the assessment visits both ‘comfortable’ and ‘enjoyable’. All surveyed key staff members reported that they would recommend ELATE to their colleagues in other aged care facilities and rated their overall satisfaction with ELATE as high.

The study received 54 referrals; however, only 16 were eligible for the study. The most common reason for ineligibility was not meeting depression criteria. This finding was unexpected given that residents were referred by staff based on their subjective opinion or resident file depression scores. The low eligibility rate may have been due to several factors such as file depression scores no longer representing the residents' current emotional state, staff misunderstanding of the presentation of depressive symptoms in older adults or residents not wishing to disclose symptoms when screened by the research assistant. Previous research has found low diagnostic accuracy of facility staff‐administered depression scales in Australia (Jeon et al., 2015). Therefore, while staff pre‐screening and subjective opinion may be useful referral tools, screening residents for depression is essential for determining study eligibility.

The study uptake rate of 93.7% was above the 72.9% rate observed in similar studies (Chan et al., 2021). All but one eligible resident consented to the study. The recruitment procedures may have contributed to this result since residents consented to the screening process with the premise of joining the study. Therefore, those who were eligible after screening were likely to consent to the larger study. The high uptake rate is encouraging for future trials, suggesting that screening protocols do not deter eligible residents from participating in the study.

The family and staff component of the treatment package was not well implemented. Only one facility engaged in more than one family or staff group session, suggesting that organising multiple face to face meetings may not be feasible for many facilities. Focusing efforts on scheduling one group session may be a better approach. Online carer support groups have been found to be successful (Daynes‐Kearney & Gallagher, 2023) and may be more feasible for family carers and staff to attend. Further, none of the family or staff members nominated by participating residents attended joint sessions as planned, suggesting that such in‐person sessions may not be feasible. Resident concerns about including support members in their treatment (e.g., burden on family) should also be identified and addressed where possible during treatment.

The results of the study are promising for the novel student‐delivered treatment model in aged care. MHT treatment adherence was high with most treatment activities delivered as planned. Strategies for modifying cognitive errors were less frequently delivered, possibly due to several participants denying having distorted cognitions. MHT competency in delivering CBT principles was lower than the accepted threshold; however, similar issues with competencies have been identified in other large‐scale studies (Goldberg et al., 2020). Out of the 11 items on the CTRS, MHTs were least consistent in setting a therapeutic agenda and homework for the resident (i.e., items related to session structure, rather than therapeutic relationship skills and CBT‐specific techniques) (Goldberg et al., 2020). The omission of these structural components may have been deliberate in some circumstances. As residents did not self‐refer to the program, residents often could not identify a target problem they wanted to work on in the program and some homework activities could also not be implemented due to physical difficulties (e.g., inability to write in journal). These findings reinforce the need for close supervision of student therapists and flexibility in treatment implementation in aged care settings. A more structured treatment approach in cases where residents deny any symptoms would also be valuable in future programs.

Importantly, both residents and staff members reported a range of benefits including a positive change in residents' mood, which aligned with preliminary clinical outcome data. Staff and residents valued having someone to talk to outside the facility, which highlights the importance of external treatment programs.

### Limitations and further research

As the sample size was small and mostly female, findings cannot be generalised to the larger population. Another reason for exercising caution in interpreting the results relating to outcomes is that some of the data collection occurred during the COVID‐19 pandemic. The pilot study lasted for 12 months (August 2019–July 2020). Four of the 15 participants (all living in one RAC facility) were unable to complete the last session of treatment and the 3‐month follow‐up assessment because of limited access to the facility. It is also possible that the increased isolation because of COVID‐19 restrictions made residents value outside social contact more, leading to amplified perceived benefits of therapy and high acceptability ratings.

This pilot focused on feasibility and acceptability rather than clinical effectiveness, and mild baseline symptoms likely limited scope for improvement. Despite this, the preliminary findings do suggest modest improvements in depression and anxiety. However, observed small‐to‐moderate effect sizes should be interpreted cautiously given the pilot design and limited sample. A larger sample size is required to examine the statistical and clinical significance of such changes and the mechanisms underlying such outcomes.

This pilot study found that the ELATE program is both feasible and acceptable for older adults in residential aged care. High uptake, strong engagement and positive feedback from residents and staff indicate its potential as a student‐delivered systemic approach. Although clinical improvements were modest, the program provided valued social support during COVID‐19 restrictions, highlighting its role in reducing isolation. These findings support further research through a larger trial to evaluate the effectiveness of ELATE as a practical and scalable approach to integrating mental health services into aged care settings and creating structured training pathways for psychology students to strengthen the future workforce.

The findings of the current pilot warrant a larger randomised controlled trial to evaluate the effectiveness of a systemic student‐delivered approach to treating depression and anxiety in RAC.

## AUTHOR CONTRIBUTIONS

**Joanna M. Waloszek:** Conceptualization; investigation; writing – original draft; writing – review and editing; formal analysis; project administration; data curation; methodology. **Sofie Dunkerley:** Conceptualization; investigation; methodology; writing – review and editing; project administration. **Sunil Bhar:** Conceptualization; investigation; funding acquisition; writing – review and editing; supervision. **Deborah Koder:** Investigation; writing – review and editing; supervision; project administration. **Tanya E. Davison:** Conceptualization; writing – review and editing; supervision; funding acquisition. **Penelope Schofield:** Conceptualization; funding acquisition; writing – review and editing; supervision. **Steven Quinn:** Funding acquisition; writing – review and editing; methodology; formal analysis. **Julie Ratcliffe:** Funding acquisition; writing – review and editing. **Mark Silver:** Writing – review and editing; project administration; investigation. **Jennifer Linossier:** Writing – review and editing; project administration; investigation. **Rebecca Collins:** Writing – review and editing; project administration; investigation. **Rachel Milte:** Writing – review and editing; supervision.

## CONFLICT OF INTEREST STATEMENT

There are no conflicts of interest to declare.

## Supporting information


Table S1.
**Table S2**.
**Table S3**.

## Data Availability

The data that support the findings of this study are available on request from the corresponding author. The data are not publicly available due to privacy or ethical restrictions.
