# Supplementary material for: Cognitive behavioural therapy for older adults with depressive symptoms living in residential aged care: A pilot study of a systemic treatment model
Source: Psychol Psychother. 2026 Apr 30;99(3):1141–56. doi: 10.1111/papt.70075 (PMC13456375; doi:10.1111/papt.70075)
Supplement: Supplementary file 1 [file PAPT-99-1141-s001.docx]

**Supplementary Table 1**

*Family and Friends Group Sessions*

| Module | Aim |
| --- | --- |
| 1. Supporting and caring for a person with anxiety and depression | Key concepts in mental health; signs and symptoms of depression and anxiety; different approaches for helping resident manage anxiety and depression; ways of getting assistance; introduction to ELATE program. |
| 2. Relationships | Discussion of the impact of depression and anxiety of relationships, identity, expression of intimacy and communication. Recognising the carer relationship as a "dyad". Acknowledge, express and examine "negative emotions" such as frustration, guilt, despair, resentment and "positive emotions" such as joy and love. Discuss strategies to manage conflicting emotions. |
| 3. Self-care | Introduce and discuss self-care strategies including mindfulness, stress management, accepting help, respite, maintaining hobbies |
| 4. Creative ways to care | Share ideas and discover different activities such as games, puzzles, music, life stories, dance to increase quality of life of resident and carer. |
| 5. Resources and where to from here | Discuss how assistance can be continued, and where further information can be gained |

**Supplementary Table 2**

*Staff Group Sessions*

| Module | Aim |
| --- | --- |
| 1 Staff training meeting | Understanding the context of depression and anxiety; Explaining the ELATE approach including role of staff; Discuss impact of mental health issues on relationships and behaviour; Discuss ways of developing partnerships and resolving issues with carers; Understanding other mental health services, what they offer and how to refer; Dealing with the impact of depression; Self-care; Support to staff- what resources are available; Sharing of experiences. |
| 2-5 Collaborative consultation meetings | Discuss interventions offered to residents. Discuss constructive approaches to helping residents with specific issues related to depression and anxiety. Acknowledge the emotional impact of working with residents and their families/carers on staff. Sharing of experiences. |

**Supplementary Table 3**

*Sample Quotes from Residents and Staff about Treatment*

(a) Social benefits

*“I enjoyed talking + having the company of a person on a regular basis”*

*“Talking to someone. Having a visitor was nice” (resident)*

*“Not really sure. I didn't think I had problems to start with but very much enjoyed.” (resident)*

(b) Mental wellbeing

*“Helped to face the situation I was in. Being in aged care, have come to some sort of acceptance. Helped me with this.” (resident)*

*“Helped with concentration, relaxation and a “few fiddly little things” (panic attacks). Learned how manage them.” (resident)*

(c) Cognitive benefits

*“Enjoyed talking to person from the “outside”. Rhonda helped her remember things from the past, which she liked. Good for her memory and made her happy.” (staff)*
